# Zerumbone-Induced Analgesia Modulated via Potassium Channels and Opioid Receptors in Chronic Constriction Injury-Induced Neuropathic Pain

**DOI:** 10.3390/molecules25173880

**Published:** 2020-08-26

**Authors:** Banulata Gopalsamy, Jasmine Siew Min Chia, Ahmad Akira Omar Farouk, Mohd Roslan Sulaiman, Enoch Kumar Perimal

**Affiliations:** 1 Department of Biomedical Sciences, Faculty of Medicine and Health Sciences, Universiti Putra Malaysia, Serdang 43400, Selangor, Malaysia; banulata@upm.edu.my (B.G.); ahmadakira@upm.edu.my (A.A.O.F.); mrs@upm.edu.my (M.R.S.); 2 Centre for Community Health Studies, Faculty of Health Sciences, Universiti Kebangsaan Malaysia, Kuala Lumpur 50300, Malaysia; jasminecsm@ukm.edu.my; 3 Australian Research Council Centre of Excellence for Nanoscale BioPhotonics, University of Adelaide, Adelaide 5000, Australia

**Keywords:** zerumbone, chronic constriction injury (CCI), allodynia, hyperalgesia, potassium channels, opioid receptors

## Abstract

Zerumbone, a monocyclic sesquiterpene from the wild ginger plant *Zingiber zerumbet* (L.) Smith, attenuates allodynia and hyperalgesia. Currently, its mechanisms of action in neuropathic pain conditions remain unclear. This study examines the involvement of potassium channels and opioid receptors in zerumbone-induced analgesia in a chronic constriction injury (CCI) neuropathic pain mice model. Male Institute of Cancer Research (ICR) mice were subjected to CCI and behavioral responses were tested on day 14. Responses toward mechanical allodynia and thermal hyperalgesia were tested with von Frey’s filament and Hargreaves’ tests, respectively. Symptoms of neuropathic pain were significantly alleviated following treatment with zerumbone (10 mg/kg; intraperitoneal, i.p.). However, when the voltage-dependent K^+^ channel blocker tetraethylammonium (TEA, 4 mg/kg; i.p.), ATP-sensitive K^+^ channel blocker, glibenclamide (GLIB, 10 mg/kg; i.p.); small-conductance Ca^2+^-activated K^+^ channel inhibitor apamin (APA, 0.04 mg/kg; i.p.), or large-conductance Ca^2+^-activated K^+^ channel inhibitor charybdotoxin (CHAR, 0.02 mg/kg; i.p.) was administered prior to zerumbone (10 mg/kg; i.p.), the antiallodynic and antihyperalgesic effects of zerumbone were significantly reversed. Additionally, non-specific opioid receptors antagonist, naloxone (NAL, 10 mg/kg; i.p.), selective µ-, δ- and κ-opioid receptor antagonists; β-funaltrexamine (β-FN, 40 mg/kg; i.p.), naltrindole (20 mg/kg; s.c.), nor-binaltorphamine (10 mg/kg; s.c.) respectively attenuated the antiallodynic and antihyperalgesic effects of zerumbone. This outcome clearly demonstrates the participation of potassium channels and opioid receptors in the antineuropathic properties of zerumbone. As various clinically used neuropathic pain drugs also share this similar mechanism, this compound is, therefore, a highly potential substitute to these therapeutic options.

## 1. Introduction

Neuropathic pain occurs following a disease or injury to the peripheral and central nervous system. This chronic pain condition remains a therapeutic challenge in clinical settings, as modern therapies are only partially effective. Various biochemical and pathophysiological changes occur following neural damage, which leads to a morphological and functional adaptation of the nervous system to external stimuli. This adaptation plays an essential role in the commencement and maintenance of pain symptoms. Even though this chronic pain state is mediated by both peripheral and central mechanisms, the pathological overexcitability of nociceptive afferents is often the trigger. Factors that lead to peripheral sensitization include sprouting of sympathetic nerves [1], inflammatory mechanisms [2], and altered activity or expression of various proteins that are related to neuronal excitability [3].

One common and consistent feature of neuropathic remodeling that occurs within the degenerating peripheral nociceptors and occasionally in the non-nociceptive afferents is the downregulation of the pool of K^+^ channels. Suppression in K^+^ channel pools is present not only in neuropathic pain conditions, but also in inflammatory and cancer pain [4]. Injured axons undergo Wallerian degeneration and demyelination of myelinated axons, whereby their functions are immediately disrupted. The distal axonal segments gradually degenerate and slowly become unexcitable. Following demyelination, Schwann cells produce new myelin sheaths as a repairing process in the peripheral nervous system. However, the architecture of these re-myelinated nerve fibers is different than normal nerve fibers [5]. Ion channels are differentially expressed due to an upsurge in the nodes number per unit length, as the number of myelin lamella is decreased. The regions that were initially internodal, having a lower density of ion channels, then have new nodes of Ranvier with denser ion channels; this density is essential for saltatory conduction [6]. Furthermore, the irregular re-myelination process may mask, block, or hide paranodal K^+^ channels, leading to the suppression of the channel function and at the same time making them resistant to drugs.

The downregulation of K^+^ channel pools has also been successfully modelled across various animal models of neuropathic pain [7,8]. Therapeutic strategies aim to activate K^+^ currents in neurons, as they are able to provide an antiexcitatory effect with no regard for the source that causes overexcitation. Considering that K^+^ channels are essential in normal nerve conductivity, a potent drug should be able to “reset” afferent excitability to a higher threshold and restore normal sensitivity. Pharmacologically, enhancement of K^+^ channels by the use of openers or enhancers has been recently identified and optimized to validate the potential of this approach as a pain treatment [9].

First-line treatments for neuropathic pain comprise tricyclic antidepressants and antiepileptics. Controlled-release opioid analgesics are often regarded as the second- or third-line treatments for moderate to severe pain and will only be prescribed if the first-line analgesic options have been exhausted [10]. Nevertheless, opioids are also prescribed as first-line treatments in certain circumstances. This is due to the effectiveness reported in some randomized clinical trials involving patients with different types of neuropathic pain [11]. Recommendations for the treatments are individualized based on the drugs’ efficacy, accessibility, side-effect profile, as well as cost-effectiveness. Opioids such as tramadol, morphine [12], methadone, and oxycodone [12,13] are the usual treatments for neuropathic pain in clinical settings.

There are a few drawbacks to the use of opioids—mainly that they often involve health complications such as sedation, dependence, dizziness, vomiting, nausea, constipation, and respiratory depression [14]. In addition, repeated or prolonged opioid administration leads to tolerance to a particular dose, resulting in a higher dosage being required to achieve the same pain relief effect [15]. Long-term opioid usage could also cause addiction, triggering compulsive drug-seeking behavior. Moreover, the discontinuation of opioid therapy results in severe withdrawal effects, which usually occur in patients who have developed tolerance [16]. Therefore, drugs derived from natural products that are able to provide substantial pain relief with fewer side effects might be preferred.

Zerumbone is an active compound isolated from the wild ginger plant, *Zingiber zerumbet* (L.) Smith. This plant is native to Southeast Asia and mainly grows in tropical and subtropical regions [17]. Ginger plants have been reported for their vast medicinal properties and have been used since earlier times as folkloric medicine [18] to treat minor diseases and ailments, such as indigestion, stomach upset, colic, cramp, morning sickness, fever, congestion, sore throat, nausea, asthma, toothache, fracture, swelling, diabetes, rheumatism, and arthritis [19,20,21]. Therefore, active compounds of this plant have been isolated and studied for their properties in recent years. Scientific testing of the possible pain relief effects of zerumbone have proved that it effectively inhibited pain in models of nociception [22,23] and inflammation [24]. Interestingly, zerumbone also attenuated allodynia and hyperalgesia in a mice model of neuropathic pain [25,26,27,28].

Zerumbone (2,6,9,9-tetramethyl-[2*E*,6*E*,10*E*]-cycloundeca-2,6,10-trien-1-one) is a monocyclic sesquiterpene with three double bonds (two conjugated and one isolated) and a conjugated carbonyl group in an 11-membered ring structure [29]. A wide array of molecular targets have been reported in existing literature on this the α,β-unsaturated, carbonyl-based compound, which has great potential for cancer and nociceptive treatments [30,31]. Recently, Hwang et al. [32] reported on the pharmacokinetic properties of zerumbone, which possesses good water solubility with blood–brain barrier and central nervous system (CNS) permeability values using absorption, distribution, metabolism, excretion, toxicity (ADMET) simulation. Using in silico methods, zerumbone has shown binding capacity to several proteins and receptor sites [33,34]. Despite the vast amount of literature on zerumbone’s characteristics, interactions of this compound with potassium channels and opioid receptors remain unknown. Based on docking analysis, the α,β-unsaturated carbonyl scaffold is the main force responsible for zerumbone’s therapeutic effects [32,35,36].

To further understand the exact underlying mechanism of this compound, we aimed to investigate if zerumbone’s actions involve potassium channels and opioid receptors in a chronic constriction injury (CCI)-induced mice model of neuropathic pain.

## 2. Results

### 2.1. Involvement of Voltage-Dependent K^+^ Channels in Zerumbone’s Antiallodynic and Antihyperalgesic Effects

**Figure 1:**
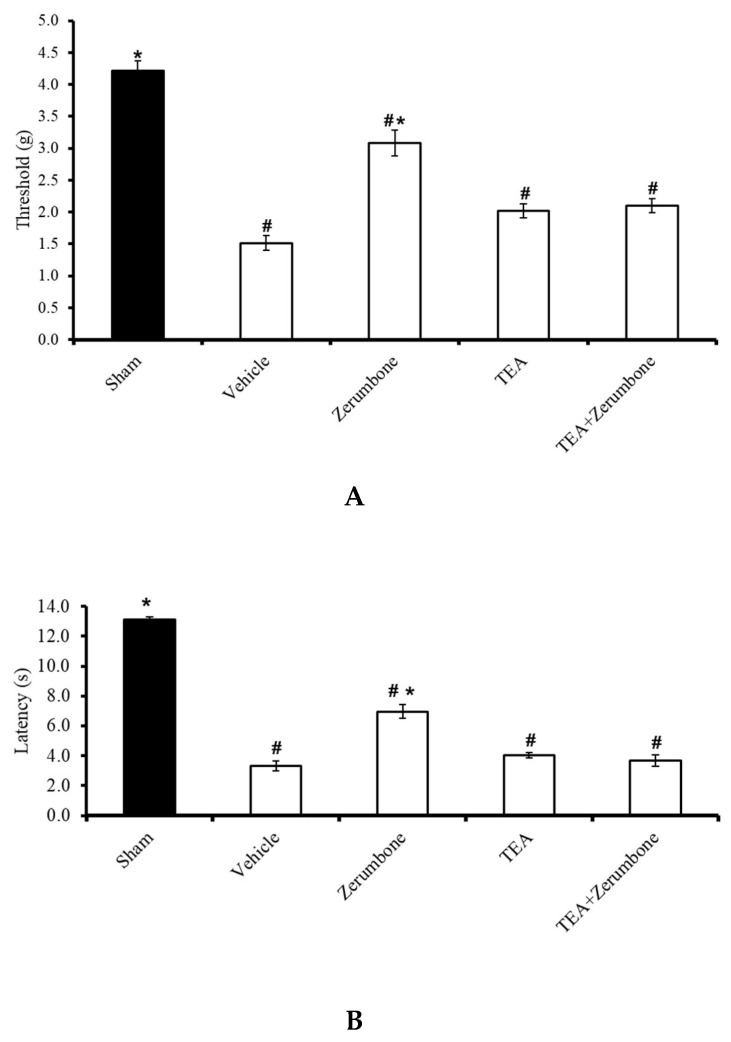
Effect of zerumbone (10 mg/kg; i.p.) and pre-treatment with a voltage-dependent K^+^ channel blocker (tetraethylammonium, TEA; 4 mg/kg; i.p.) on the responses toward (**A**) mechanical allodynia and (**B**) thermal hyperalgesia on chronic constriction injury (CCI)-induced neuropathic pain in mice. Each column represents the mean ± SEM; n = 8 mice per group. Note: ^#^ significantly different (*p* ≤ 0.05) than sham group; * significantly different (*p* ≤ 0.05) than vehicle group (one-way ANOVA followed by Tukey’s post hoc test).

The involvement of voltage-dependent K^+^ channels in the antineuropathic properties of zerumbone was investigated by blocking the channels with a voltage-dependent K^+^ channel blocker, tetraethylammonium (TEA). Pre-treatment the animals (n = 8) with TEA (4 mg/kg; i.p.) prior to zerumbone significantly reversed the antiallodynic effect of zerumbone (10 mg/kg; i.p.) (*p* ≤ 0.05) (Figure 1A). Similarly, pre-treatment with TEA (n = 8, 4 mg/kg; i.p.) also reversed the antihyperalgesic effect of zerumbone (10 mg/kg; i.p.) (*p* ≤ 0.05) (Figure 1B). Treatment with TEA alone (n = 8) did not elicit any effect on the withdrawal threshold or latency in either test.

### 2.2. Involvement of ATP-Sensitive K^+^ Channels in Zerumbone’s Antiallodynic and Antihyperalgesic Effects

**Figure 2:**
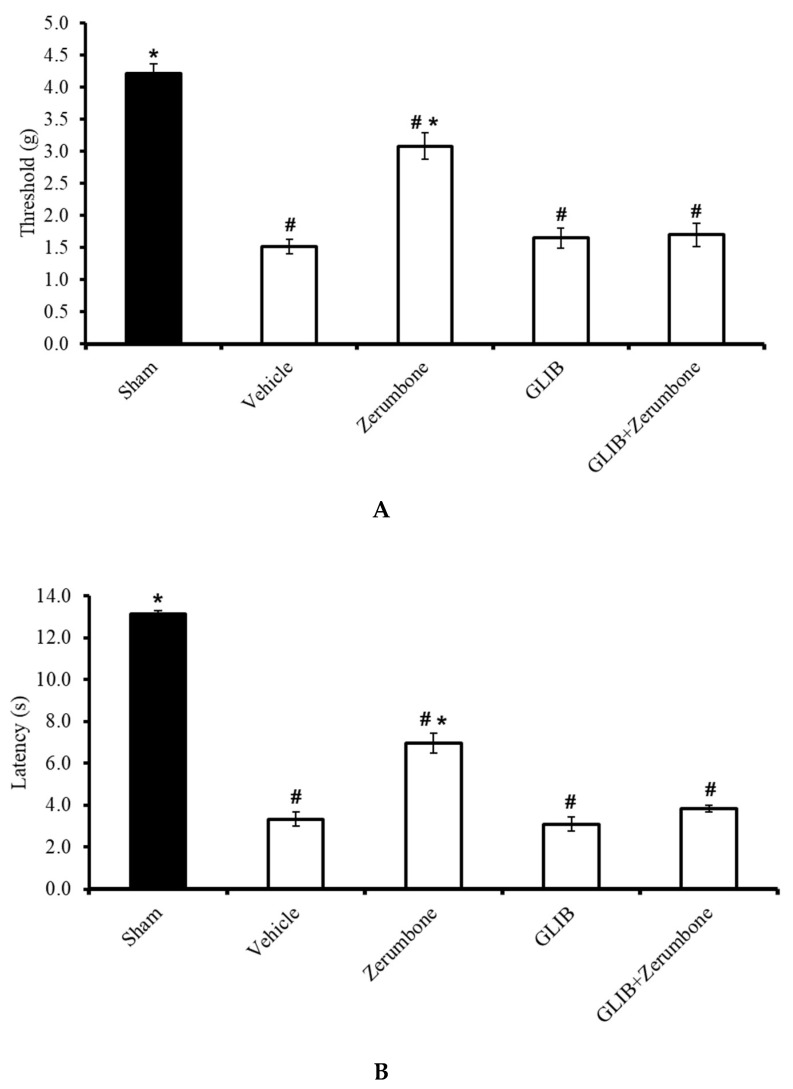
Effect of zerumbone (10 mg/kg; i.p.) and pre-treatment with an ATP-sensitive K^+^ channel blocker (glibenclamide, GLIB; 10 mg/kg; i.p.) on the responses toward (**A**) mechanical allodynia and (**B**) thermal hyperalgesia on CCI-induced neuropathic pain in mice. Each column represents the mean ± SEM; n = 8 mice per group. Note: ^#^ significantly different (*p* ≤ 0.05) than sham group; * significantly different (*p* ≤ 0.05) than vehicle group (one-way ANOVA followed by Tukey’s post hoc test).

The involvement of ATP-sensitive K^+^ channels in the ability of zerumbone to induce analgesia was investigated by pre-treating the animals with an ATP-sensitive K^+^ channel antagonist, glibenclamide (GLIB). GLIB (10 mg/kg; i.p.) significantly reversed the antiallodynic effect (n = 8) (Figure 2A) and antihyperalgesic (n = 8) (Figure 2B) effects of zerumbone (10 mg/kg; i.p.) (*p* ≤ 0.05). Administration of the antagonist alone did not elicit any effect on this mice model.

### 2.3. Involvement of Small- and Large-Conductance Ca^2+^-Activated K^+^ Channels in Zerumbone-Induced Antiallodynia and Antihyperalgesia

**Figure 3:**
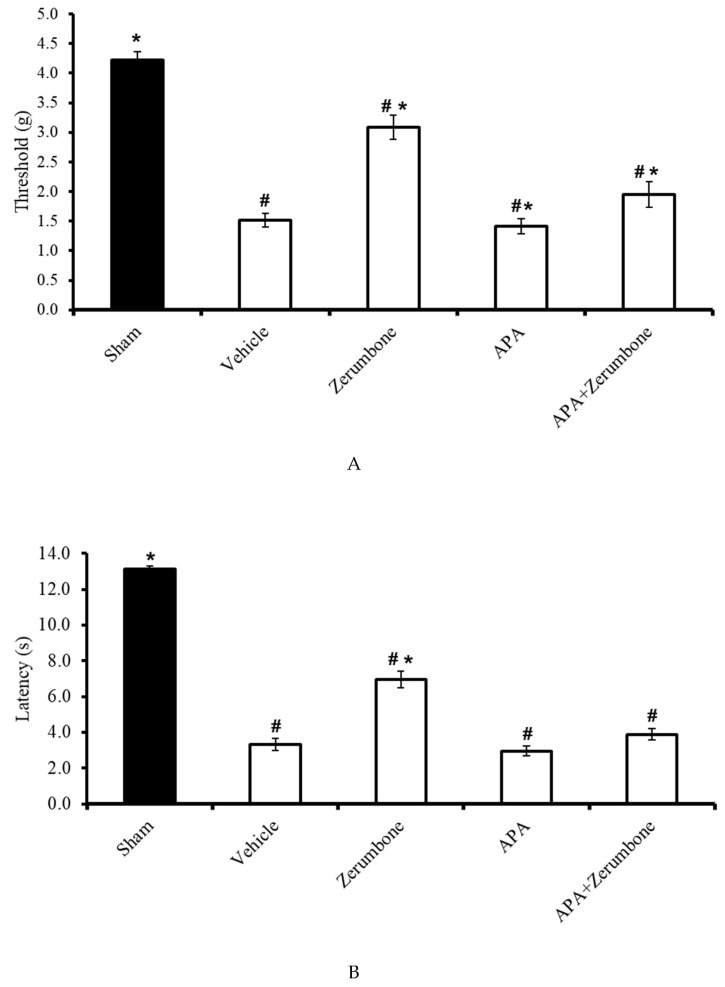
Effect of zerumbone (10 mg/kg; i.p.) and pre-treatment with a small-conductance Ca^2+^-activated K^+^ channel inhibitor (apamine, APA; 0.04 mg/kg; i.p.) on the responses toward (**A**) mechanical allodynia and (**B**) thermal hyperalgesia on CCI-induced neuropathic pain in mice. Each column represents the mean ± SEM; n = 8 mice per group. Note: ^#^ significantly different (*p* ≤ 0.05) than sham group; * significantly different (*p* ≤ 0.05) than vehicle group (one-way ANOVA followed by Tukey’s post hoc test).

The involvement of small-conductance Ca^2+^-activated K^+^ channels in the antineuropathic properties of zerumbone was investigated using a selective small-conductance Ca^2+^-activated K^+^ channel inhibitor, apamin (APA). APA (0.04 mg/kg; i.p.) was administered prior to zerumbone treatment, while the antiallodynic (n = 8) (Figure 3A) and antihyperalgesic (n = 8) (Figure 3B) effects of zerumbone (10 mg/kg; i.p.) (*p* ≤ 0.05) were absent. Treatment with APA (0.04 mg/kg; i.p.) alone did not elicit any effect on the animal’s behavioral responses.

**Figure 4:**
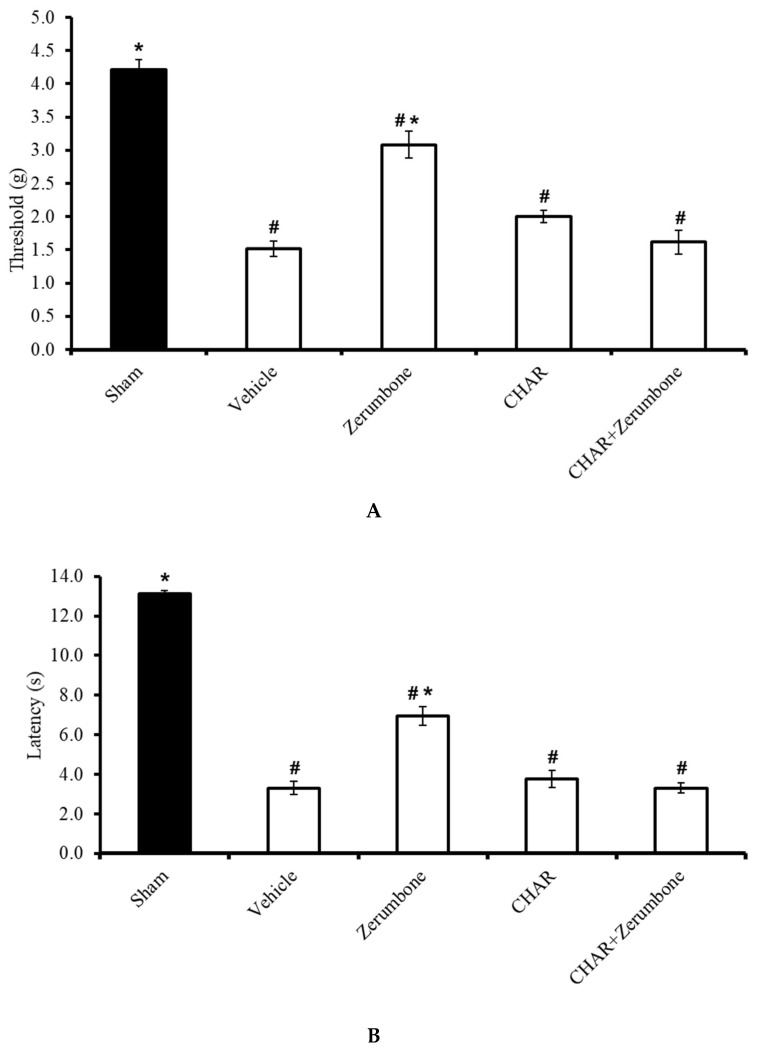
Effect of zerumbone (10 mg/kg; i.p.) and pre-treatment with a large-conductance Ca^2+^-activated K^+^ channel inhibitor (charybdotoxin, CHAR; 0.02 mg/kg; i.p.) on the responses toward (**A**) mechanical allodynia and (**B**) thermal hyperalgesia on CCI-induced neuropathic pain in mice. Each column represents the mean ± SEM; n = 8 mice per group. Note: ^#^ significantly different (*p* ≤ 0.05) than sham group; * significantly different (*p* ≤ 0.05) than vehicle group (one-way ANOVA followed by Tukey’s post hoc test).

The large-conductance Ca^2+^-activated K^+^ channel inhibitor charybdotoxin (CHAR; 0.02 mg/kg; i.p.) was administered prior to zerumbone (10 mg/kg; i.p.) to investigate whether the action of zerumbone is carried out via large-conductance Ca^2+^-activated K^+^ channels. The results show significant (*p* ≤ 0.05) reversal of the antiallodynic (n = 8) (Figure 4A) and antihyperalgesic (n = 8) (Figure 4B) effect elicited by zerumbone alone, demonstrating the role of large-conductance Ca^2+^-activated K^+^ channels in zerumbone’s properties of attenuating neuropathic pain symptoms.

### 2.4. Involvement of Non-Selective Opioid Receptors

**Figure 5:**
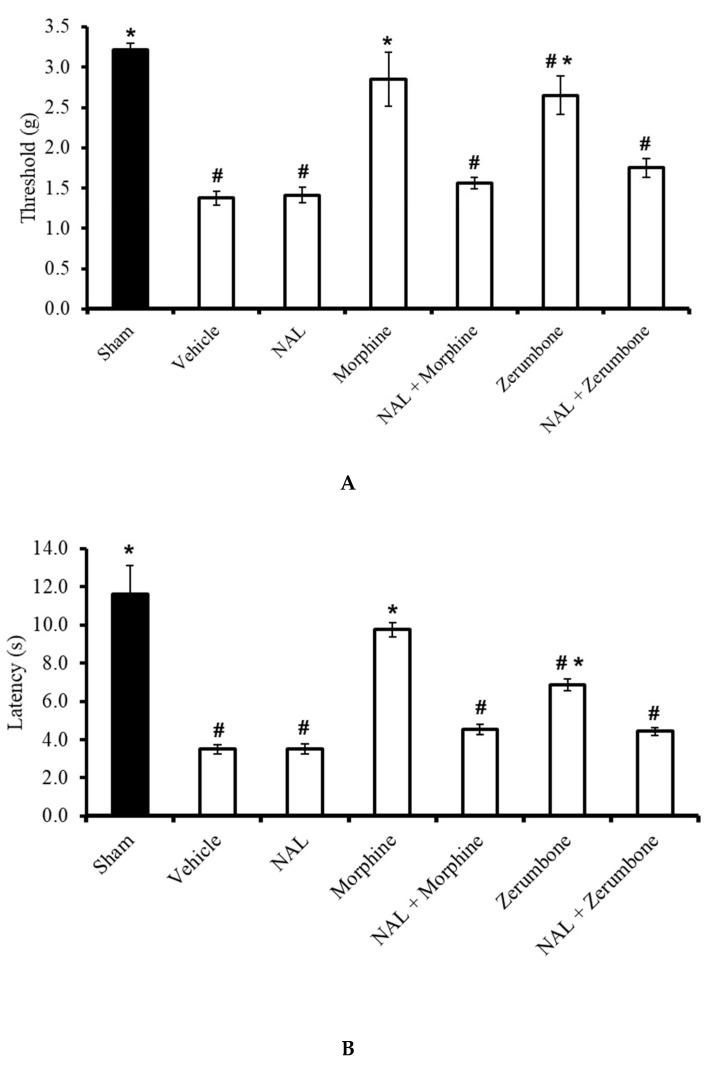
Effects of zerumbone (10 mg/kg; i.p.) and morphine (10 mg/kg; i.p.) and pre-treatment with a non-selective opioid receptor antagonist (naloxone, NAL; 10 mg/kg; i.p.) on the responses toward (**A**) mechanical allodynia and (**B**) thermal hyperalgesia on CCI-induced neuropathic pain in mice. Each column represents the mean ± SEM; n = 8 mice per group. Note: ^#^ significantly different (*p* ≤ 0.05) than sham group; * significantly different (*p* ≤ 0.05) than vehicle group (one-way ANOVA followed by Tukey’s post hoc test).

The antiallodynic effects observed in the zerumbone (10 mg/kg; i.p.)-treated group was absent when the animals were pre-treated with naloxone (NAL; 10 mg/kg; i.p.) before administering zerumbone (10 mg/kg; i.p.) (*p* ≤ 0.05) (Figure 5A). Similarly, the outcome of the Hargreaves’ test shows that the administration of NAL (10 mg/kg; i.p.) before zerumbone (10 mg/kg; i.p.) treatment caused a complete reversal (*p* ≤ 0.05) of zerumbone’s antihyperalgesic effect (Figure 5B). It is important to note that NAL (10 mg/kg; i.p.) alone does not exhibit any significant (*p* > 0.05) effect on CCI mice. In both tests, the antiallodynic and antihyperalgesic effects of morphine in CCI-induced mice were also reversed by pre-treatment of the non-selective opioid receptor blocker.

### 2.5. Involvement of Selective µ-Opioid Receptors

**Figure 6:**
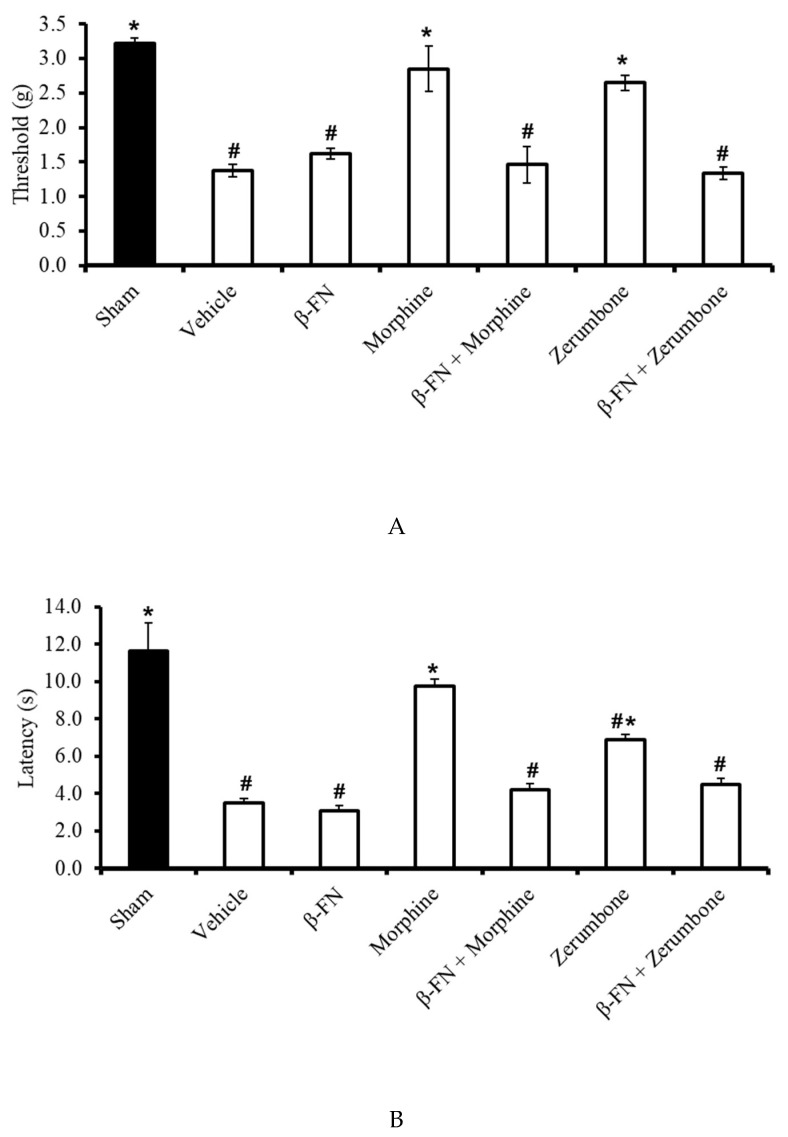
Effect of zerumbone (10 mg/kg; i.p.) and pre-treatment with a selective µ-opioid receptor antagonist (β-funaltrexamine, β-FN; 40 mg/kg; s.c.) on the responses toward (**A**) mechanical allodynia and (**B**) thermal hyperalgesia on CCI-induced neuropathic pain in mice. Each column represents the mean ± SEM; n = 8 mice per group. Note: ^#^ significantly different (*p* ≤ 0.05) than sham group; * significantly different (*p* ≤ 0.05) than vehicle group (one-way ANOVA followed by Tukey’s post hoc test).

The participation of µ-opioid receptor subtypes was investigated by blocking the receptors with a selective µ-opioid antagonist, β-funaltrexamine (β-FN). Pre-treatment with β-FN (40 mg/kg; subcutaneous, s.c.) prior to zerumbone significantly reversed the antiallodynic effect of zerumbone (10 mg/kg; i.p.) (*p* ≤ 0.05) (Figure 6A). Similarly, pre-treatment with β-FN (40 mg/kg; s.c.) also reversed the antihyperalgesic effect of zerumbone (10 mg/kg; i.p.) (*p* ≤ 0.05) (Figure 6B).

### 2.6. Involvement of Selective δ-Opioid Receptors

**Figure 7:**
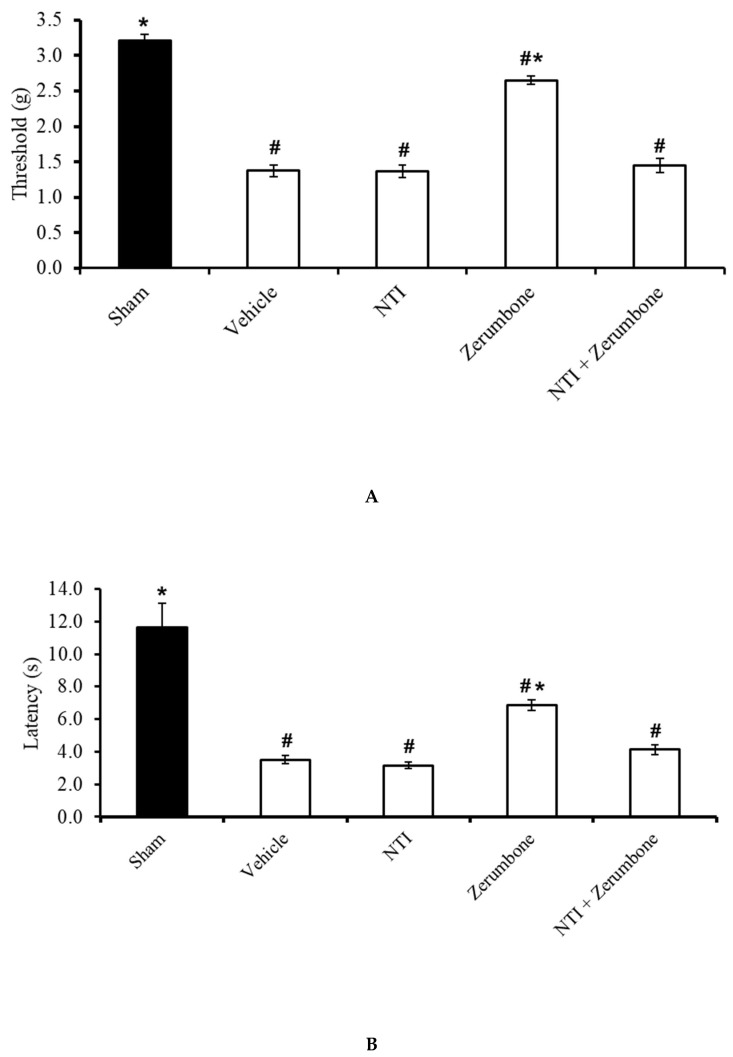
Effect of zerumbone (10 mg/kg; i.p.) and pre-treatment with a selective δ-opioid receptor antagonist (naltrindole, NTI; 20 mg/kg; s.c.) on the responses toward (**A**) mechanical allodynia and (**B**) thermal hyperalgesia on CCI-induced neuropathic pain in mice. Each column represents the mean ± SEM; n = 8 mice per group. Note: ^#^ significantly different (*p* ≤ 0.05) than sham group; * significantly different (*p* ≤ 0.05) than vehicle group (one-way ANOVA followed by Tukey’s post hoc test).

The involvement of δ-opioid receptors in the action of zerumbone was investigated by pre-treating the animals with a selective δ-opioid subtype antagonist, naltrindole (NTI). Pre-treatment with NTI (20 mg/kg; s.c.) prior to zerumbone significantly reversed the antiallodynic (Figure 7A) and antihyperalgesic (Figure 7B) effects of zerumbone (10 mg/kg; i.p.) (*p* ≤ 0.05).

### 2.7. Involvement of Selective κ-Opioid Receptors 

**Figure 8:**
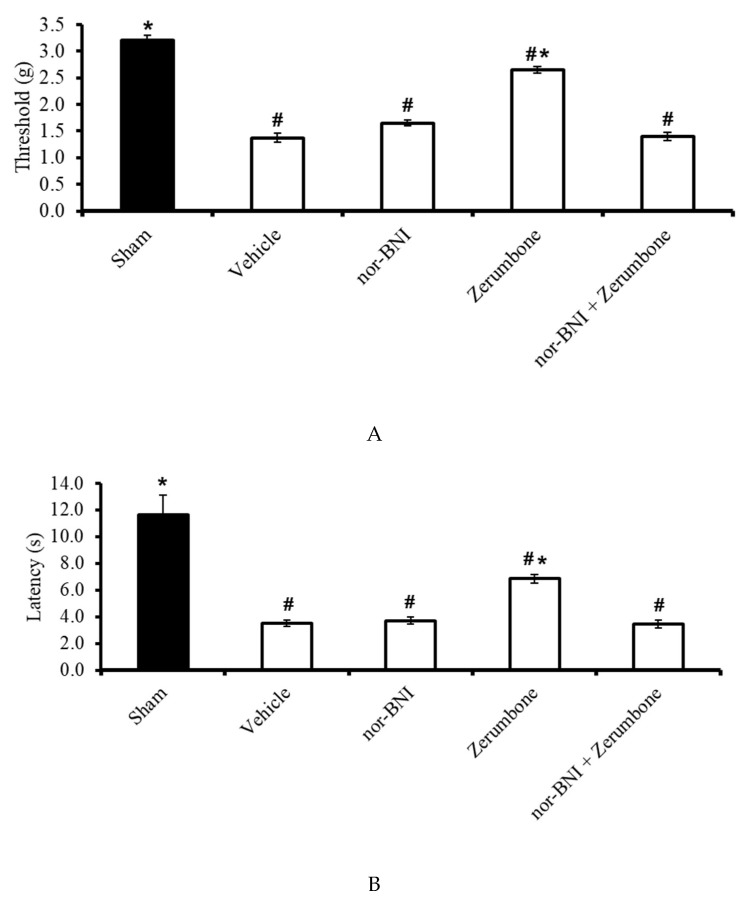
Effect of zerumbone (10 mg/kg; i.p.) and pre-treatment with a selective κ-opioid receptor antagonist (nor-binaltorphamine, nor-BNI; 10 mg/kg; s.c.) on the responses toward (**A**) mechanical allodynia and (**B**) thermal hyperalgesia on CCI-induced neuropathic pain in mice. Each column represents the mean ± SEM; n = 8 mice per group. Note: ^#^ significantly different (*p* ≤ 0.05) than sham group; * significantly different (*p* ≤ 0.05) than vehicle group (one-way ANOVA followed by Tukey’s post hoc test).

The involvement of κ-opioid receptors in the antineuropathic properties of zerumbone were investigated using a selective κ-opioid subtype antagonist, nor-binaltorphimine (nor-BNI; 10 mg/kg; s.c.). The nor-BNI was administered prior to zerumbone treatment and the antiallodynic (Figure 8A) and antihyperalgesic (Figure 8B) effects of zerumbone (10 mg/kg; i.p.) (*p* ≤ 0.05) were absent. Treatment with nor-BNI (10 mg/kg; s.c.) alone did not elicit any effect on the animal’s behavioral responses.

### 2.8. Rota Rod Assay 

**Figure 9:**
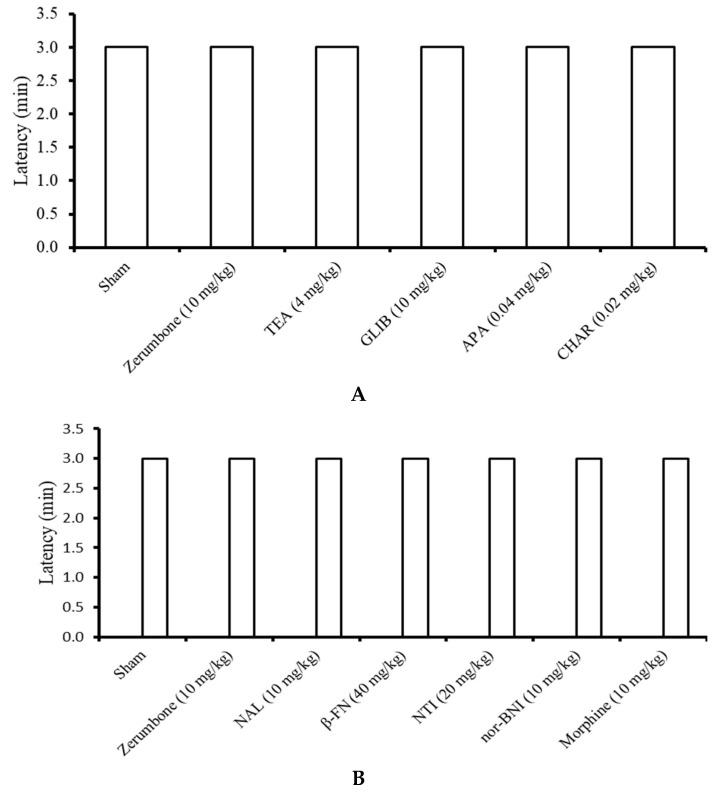
Effects of the treatments on the rota rod performance in mice following treatment with zerumbone, (**A**) potassium channel antagonists, and (**B**) opioid receptor antagonists. (TEA: Tetraethylammonium; GLIB: Glibeclamide; APA: Apamine; CHAR: Charybdotoxin; NAL: Naloxone; β-FNI: β-funaltrexamine; NTI: Naltrindole; nor-BNI: nor-binaltorphamine). Values represent the means ± SEM; n = 8 mice per group (one-way ANOVA followed by Tukey’s post hoc test).

All the mice in the sham, zerumbone (10 mg/kg; i.p.), TEA (4 mg/kg; i.p.), GLIB (10 mg/kg; i.p.), APA (0.04 mg/kg; i.p.), CHAR (0.02 mg/kg; i.p.), NAL (10 mg/kg; i.p.), β-FN (40 mg/kg; i.p.), NAL (20 mg/kg; s.c.), nor-BNI (10 mg/kg; s.c.), and morphine (10 mg/kg; i.p.) groups were able to survive on the rota rods throughout the period of three minutes (n = 8) (Figure 9).

## 3. Discussion

The involvement of K^+^ channels, specifically the K_V_, K_ATP_, SK_Ca_, and BK_Ca_ channels, were demonstrated in zerumbone’s action of inducing analgesia in the CCI model of neuropathic pain. This is due to the reversal of the antiallodynic and antihyperalgesic effects exhibited by zerumbone (10 mg/kg; i.p.) following pre-treatment with channel blockers or inhibitors. The respective inhibitors specifically deterred zerumbone’s action on those channels, meaning zerumbone failed to lower the pain threshold or latency values. The rota rod assay ensured that the outcome was entirely a behavioral response and not a consequence of impaired motor function, a possible sedative effect of the treatments, or a suppression of general behavior [37,38], as all mice were able to survive on the rota rod for the entire three minutes without falling or rolling over.

Voltage-gated potassium channels (K_V_) channels in myelinated axons are present in the paranodal, internodal, and even the juxtaparanodal regions. However, they are generally absent in the nodal regions in mammalian nerves [39]. K_V_ channels are also present in unmyelinated axons, as well as in distinct populations of dorsal root ganglion (DRG) neurons, which influence neuronal excitability following pharmacological blockade [40]. In the case of nerve injury, myelin sheaths will be disrupted or removed, leading to conduction blockage. The blockage is due to an increase in membrane capacitance and decline in membrane resistance. K^+^ channels at juxtaparanodes are usually electrically isolated but are exposed or appear uncovered during demyelination, reducing neuronal excitability. Therefore, pharmacological compounds that block K_V_ channels improve conduction in those demyelinated axons [39].

The opening of K_V_ channels could be blocked by TEA, a small ion that binds to the inner and outer sites of the channels. Blockade at the internal mouth of the channel pores is voltage-dependent but blockade at the outer mouth is almost voltage independent [41]. In this study, intraperitoneal administration of TEA alone at 4 mg/kg had no effect on the behavioral response. However, zerumbone’s antiallodynic and antihyperalgesic effects were absent following pre-treatment of TEA, clearly indicating the involvement of K_V_ channels in zerumbone-induced analgesia.

There are various mechanisms for drugs to carry out their functions. Drug molecules can reside in the pockets in the inner mouth of channel pores, where typical blockers reside when they interact with other molecules. These drugs displace the blockers, preventing them from acting on the channels [42]. Another interesting mechanism is when drug molecules attach themselves to the “gating hinges”, disrupting the normal function of the channel gates. Retigabine, a K_V_7 activator, uses this mechanism in its action [43]. Furthermore, drugs can also act as disinactivators and interrupt the association between α- and β-subunits, thus altering the channel behavior [44].

Many drugs and analgesics share similar mechanisms by acting as K_V_ channel openers or activators. Triaminopyridines such as flupiritne and retigabine are analgesics, which act by enhancing maximum steady-state K^+^ conductance at saturating voltages. Thermal hyperalgesia in neuropathic rats was alleviated when flupitine was injected at neuroma sites [45]. Retigabine on the other hand reduces bradykinin-induced pain [46] and carrageenan-induced hyperalgesia [47]. Fampridine, a non-selective K_V_ channel modulator, has already been established as being able to treat multiple sclerosis [48].

K_ATP_ channels can be selectively blocked by glibenclamide [49]. In this study, the involvement of K_ATP_ channels in zerumbone’s antiallodynic and antihyperalgesic effects was clearly demonstrated. Likewise, Perimal et al. [23] reported that zerumbone exhibited marked inhibition of pain against chemical models of nociception in mice, with the possible opening of K_ATP_ channels.

Clinically available drugs such as clonidine [50], 5-HT_1_ agonists [51], and morphine [52] are analgesics that are specifically mediated by K_ATP_ channels. Other blockers of potassium channels do not alter the analgesic effects of these drugs but are only reversed following pre-treatment with a selective K_ATP_ antagonist. Other drugs such as cromakalim, pinacidil [53], minoxidil [54], and nicorandil [55] have also been reported to mediate their analgesic effects via K_ATP_ channels.

A high expression of BK_Ca_ channels in the trigeminal ganglion [56], superficial dorsal horn [57], and dorsal root ganglion, DRG [58] induces antihyperalgesic effects in neuropathic pain models. However, the expression of BK_Ca_ channels was suppressed in DRG neurons following L4–L5 nerve ligation injury [58] and in the superficial dorsal horn in a partial sciatic nerve ligation model [57].

The involvement of BK_Ca_ channels in the antiallodynic and antihyperalgesic effects of zerumbone was also observed in this study, as the effects were reversed when mice were pre-treated with CHAR (0.02 mg/kg; i.p.), a selective BK_Ca_ channel inhibitor. The opening of BK_Ca_ channels in DRG neurons reduces the depolarization-evoked firing of action potentials [59]. BK_Ca_ channels are ideal cell excitability feedback regulators. This is due to BK_Ca_ channels having high conductance, whereby the duration of action potentials is shortened by BK_Ca_ channel activation. This consequently increases the rate of repolarization and reduces depolarization, leading to rapid post-polarization effects [59].

The stimulation of BK_Ca_ is subject to dual control, where it is either activated by a rise in the cytosolic Ca^2+^ concentration or by membrane depolarization. The induction of BK_Ca_ openings can be caused by intracellular free calcium alone while the domains of voltage sensors remain activated [60]. Furthermore, the induction of these channels can also be due to voltage alone, indicating that the dual control of these channels could function either synergistically or via independent mechanisms [61].

The large-conductance BK_Ca_ channels are rather weakly sensitive to voltage [62]. When cytoplasmic Ca^2+^ concentrations are at resting levels, the BK_Ca_ channels can only be opened by the presence of very positive voltages. However, the voltage-dependent activation takes a leftward shift along the voltage axis towards a more negative membrane potential. An increase in the free Ca^2+^ concentration shifts the activity of these channels in the physiologic membrane potential range [61]. Membranes remain hyperpolarized when BK_Ca_ channels are in an open state during neuronal firing. This further causes an inhibitory feedback, limiting the influx of Ca^2+^ and excitability. Therefore, these channels are powerful regulators of synaptic transmission at nerve terminals [63]. Due to maladaptive pain signaling and abnormal excitability of the somatosensory system in chronic pain conditions, the role of these channels is vital, and drug targeting of these channels and their actions might be more effective.

Small-conductance SK_Ca_ channels were also involved in the antiallodynic and antihyperalgesic effects of zerumbone in this study, as the compound’s effects were reversed when mice were pre-treated with apamin (APA; 0.04 mg/kg; i.p.), a selective SK_Ca_ channel inhibitor. This outcome suggests that zerumbone enhances SK_Ca_ channel activity, leading to a substantial reduction in the sensory input. Compound (*E*)-2-(4,6-difluoro-1-indanylidene) acetamide and drugs such as chloroxazone [64] and riluzole [65] are enhancers of SK_Ca_ channel activity and are potent analgesics. The effects of resveratrol, a drug that exhibits peripheral antinociceptive activity, were reversed in the presence of APA [66].

Zerumbone also used the opioidergic pathway to elicit its analgesic effects on the neuropathic pain models. This is because the antiallodynic and antihyperalgesic effects exerted by zerumbone (10 mg/kg; i.p.) were reversed when mice were pre-treated with a non-selective opioid receptor antagonist, NAL. NAL showed no effect when administered alone. Similarly, morphine (positive control), an opiate known for its analgesic action, specifically via the µ-opioid receptor, also showed a complete reversal of its action when treated with NAL.

The opioidergic pathway plays a role in pain modulation, whereby activation of opioid receptors is implicated with pain regulation, neuroendocrine modulation, reinforcement and reward behavior, and changes in neurotransmitter release. Numerous endogenous opioid peptides such as dynorphin-A, met-enkeplin, and β-endorphin are produced by the body’s innate response to pain [67]. Opioid-mediated analgesia includes both the ascending and descending pain pathways and exerts both centrally and peripherally mediated effects [67]. Opioids act as agonists of either one or more of the three classic opioid receptors subtypes (µ, κ, and δ) to activate the pathway [68].

Activation of opioid receptors induces the pain inhibitory modulation via three main events, which are activation of the inwardly rectifying K^+^ channels, inhibition of the voltage sensitive Ca^2+^ channels, and reduction of the cyclic adenosine monophosphate (cAMP) production following the inhibition of adenylyl cyclase [69]. Conversely, opioid receptors utilize other intermediate messenger systems to activate the cascade of mitogen-activated protein kinase, phospholipase C, and large-conductance Ca^2+^-activated K^+^ channels [70]. This series of events, especially the modulation of K^+^ and Ca^2+^ channels, lowers neuronal excitability, decreases the rate of neuronal firing, and inhibits neurotransmitter release [67,71].

Opioid receptors are widely distributed in the brain [72,73,74]. Two important brain centers involved in opioid-produced antinociception are the rostral ventromedial medulla (RVM) and periaqueductal gray (PAG) of the midbrain. These centers are critical targets for both endogenous opioids and opioid pharmaceuticals. Spinally projecting neurons in the RVM are activated by cells in the PAG, which then inhibit nociceptive cells in the spinal cord [75,76]. Furthermore, high densities of opioid receptors can also be found in the hypothalamus, hippocampus [77], habenula, nucleus raphe magnus, caudate nucleus, and the spinal cord [74]. It is important to note that opioid receptors are also found in peripheral neurons, which also play a role in antinociception [67]. However, from this experimental design we could not speculate on the exact site of zerumbone’s action, as the compound was employed via the intraperitoneal route and was present systemically.

Zerumbone’s ability to attenuate thermal hyperalgesia was reversed when the mice were pre-treated with β-FN, the µ-opioid receptor antagonist. This indicates that zerumbone possibly acts as the µ-opioid receptors’ agonist and modulates pain by activating these receptors. It was reported earlier that the functional involvement of different opioid receptor subtypes and neuronal pathways differentially is influenced by the modalities of the noxious stimuli [78]. A thermal noxious stimulus responds more effectively to µ-type agonists [79], further supporting the effectiveness of zerumbone in attenuating thermal hyperalgesia. Morphine, whose analgesic effect is modulated by µ-opioid receptors, is a drug that is prescribed for the management of moderate to severe pain [80]. The clear benefits of µ-agonists in pain treatments have been reported for over 1000 years [80].

The antiallodynic and antihyperalgesic effects of zerumbone were reversed when δ-opioid receptors were blocked with a specific δ-opioid receptor antagonist, naltrindole. This clearly indicates the involvement of these receptors in zerumbone-induced analgesia. The function of δ-opioid receptors in the pain pathway was evident when δ-receptor knockout mice amplified inflammatory [81] and neuropathic [82] pain conditions. This demonstrates the presence of endogenous δ-opioid receptors’ activity in effectively reducing chronic and persistent pain. Therefore, systemically active δ-opioid receptor agonists are useful targets for chronic pain [83]. Various novel δ-agonists have been developed as preclinical model analgesics. They include NIH 11,082 [84], DL5859 [85], KNT-127 [86], and compound 8e [87].

Zerumbone when administered alone reduced the pain response towards mechanical allodynia and thermal hyperalgesia. However, when mice were pre-treated with nor-binaltorphamine, a κ-opioid receptor antagonist, the effects of zerumbone were reversed. This indicates that zerumbone also acts as a κ-opioid receptor agonist to carry out pain modulation. Nociception caused by pressure has also been reported to preferentially respond to κ-opioid receptor agonists [79], providing evidence of the properties of zerumbone, which shows a lowered response towards mechanical hyperalgesia.

Although κ-opioid agonists’ maximum effect has been reported to be weaker than µ-opioids such as morphine, researchers were still very interested in developing κ-opioid agonists. This is because κ-opioid agonists can be used for pain-relief without activating the reward pathways, which are stimulated by µ-opioids. However, κ-opioid agonists can cause problems, such as constipation, dysphoria, and diuresis [88]. Therefore, if zerumbone was able to act in the same way as κ-opioid agonists in providing analgesia without the presence of adverse effects, perhaps it would be a better option to be considered as a treatment for pain relief.

Furthermore, the activation of κ-opioid receptors directly closes Na^+^ channels [89]. This is an important feature of any drug for the treatment of neuropathic pain, as the pathophysiology of neuropathic pain shows the increased density and expression of abnormal of Na^+^ channels along the primary afferent neurons. The increased spontaneous membrane potential oscillation and alterations to the conductance of those channels reduce the firing threshold, resulting in the spontaneous activity of sensory neurons [90]. Drugs such as lidocaine, which act as sodium channel blockers, are currently used as treatments for neuropathic pain [90].

Overall, we found that zerumbone utilizes the opioidergic pathway. Natural products are known to utilize multiple receptors of varying mechanisms to exhibit their actions. Similarly, we hypothesize that zerumbone acts through multiple receptors to attenuate allodynia and hyperalgesia in neuropathic pain models. Previously, zerumbone was shown to utilize the serotonergic pathway in CCI-induced allodynia and hyperalgesia [27]. Activation of serotonergic receptors stimulates the release of opioids and gamma-aminobutyric acid, GABA, thus inhibiting transmission of nociceptive signals [91]. Therefore, we postulate that serotonin and its receptors may correlate with the opioidergic pathway for zerumbone to exhibit its antineuropathic effects.

In the study by Zulazmi et al. [26], the antiallodynic and antihyperalgesic effects of zerumbone in a neuropathic pain mouse model was postulated to act through L-arginine-nitric oxide-cGMP-K^+^ ATP channels. The NO-cGMP pathway activation and K^+^ channels have been shown to correlate [92,93]. In a recent report by the research team, the antiallodynic and antihyperalgesic effects of zerumbone were shown to interact with the noradrenergic system, TRPV1, and NMDA receptors [94]. The noradrenergic system has been shown to act synergistically with the opioidergic pathway at both spinal and supraspinal sites [95,96,97]. TRPV and NMDA receptors are primarily involved in excitatory nociceptive processing. The nociceptive action of these receptors is modulated by calcium ions [98,99]. As mentioned earlier, the opioidergic pathway reduces neuronal activity by modulating calcium and potassium channels. Therefore, current literature on the mechanisms of action of zerumbone support our current observations regarding opioid receptors.

The antagonists of potassium channels TEA, GLIB, APA, and CHAR; and opioid receptors NAL and NTI were administered 15 min prior to zerumbone treatment, but β-FN and nor-BNI were administered 24 h prior to zerumbone treatment. This is because different drugs and blockers have different targets, binding affinities, and efficacies, which take different durations to exhibit their effects [100]. The mode of drug administration also differs between groups. Drug administration via the intraperitoneal mode causes faster absorption into the blood stream compared to subcutaneous administration. Drugs are administered subcutaneously if a slower release of the drug into the vasculature is required, whereby fast administration might produce adverse effect such as respiratory depression [100]. The antagonist’s modes of administration, dose, and duration of effect were pre-tested to ensure they did not increase pain or sensitivity in the animals prior to testing on the actual experimental animals. This protocol was reported by Ming-Tatt et al. [92] and Zakaria et al. [101].

The pathophysiological changes that occur after nerve injury include altered expression and efficacy of potassium channels and opioid receptors at various sites of the pain pathway. The changes can occur in the peripheral nerves, dorsal root ganglion, spinal cord, along the ascending and descending pathways, as well as in the brain [9,102,103]. In this study, we administered zerumbone systemically to test the involvement of potassium channels and opioid receptors. However, the exact target site of zerumbone could not be determined in this study design, which is a limitation of this study. In this study, evidence was provided of the involvement of potassium and opioid receptors in zerumbone’s action solely on the behavioral outcome. However, we did not screen for the effect of zerumbone on the receptor and receptor subtype’s molecular expression along the pain pathway, which is another limitation of this study. Therefore, future studies should evaluate the underlying molecular mechanisms and narrow down the specific sites of zerumbone’s action to allow translational research into targeted forms of therapy.

The expression of opioid receptors and potassium channels was altered in CCI animals. The effects of each antagonist were confirmed by having groups treated by zerumbone alone and antagonists alone to confirm the partial contribution of each as a standard protocol for these experiments. A study by Le Guen et al. [104] reported that when opioid receptor antagonists were administered into naïve rats, behavioral changes occurred, indicating tonic activity in the endogenous opioid peptides acting on mu opioid receptors. NAL or β-FN triggered Fos-like immunoreactivity in the nucleus of the solitary tract, area postrema, rostral ventrolateral medulla, supramammillary nucleus, central nucleus of the amygdala, and the Kölliker–Fuse nucleus of the central nervous system [104,105]. NTI and nor-BNI showed no effect on naïve rats [104].

On the other hand, potassium channel blockers (TEA, APA, CHAR) reduce the mouse immobility time of intensity in forced swimming tests, an animal model for depression. These blockers produce an antidepressant-like effect by preventing hyperpolarization, leading to a higher excitatory response [106]. Hyperalgesia and antinociception also do not occur in mice when GLIB is administered alone [106,107].

When a particular drug that uses a single receptor or channel type provides a good level of analgesia, it is presumed that the summation effect of zerumbone (i.e., using all µ-, κ-, and δ-opioid receptors, as well as K_V_, KATP, BKCa, and SKCa channels) should provide better pain relief. However, this was not reflected in our outcome, as the pain threshold and latency were lower than the levels in the sham control group and in the animals that received morphine. Achieving 100% analgesia is almost unachievable, unless it is accompanied by severe sedative and nervous suppression. The pharmacodynamics of zerumbone is not fully understood, but it is known to interact with multiple pathways without causing any adverse effects. We are unable to provide a summation analysis of the individual effects of zerumbone through these experiments, as the antagonists were administered on different groups of animals. Therefore, we suggest that future studies could aim to characterize the binding properties of zerumbone in opioid receptors and potassium channels.

## 4. Materials and Methods

### 4.1. Preparation of Zerumbone

Zerumbone was extracted from the rhizomes of *Zingiber zerumbet* as outlined by Perimal et al. [23]. Rhizomes of *Z. zerumbet* were obtained from the Chow Kit wet market in Kuala Lumpur. Mr. Shamsul Khamis, a resident botanist at Institute of Bioscience, Universiti Putra Malaysia, identified and confirmed the plant species, then a sample specimen was inserted at the herbarium of the Laboratory of Natural Products, Institute of Bioscience, Universiti Putra Malaysia, with the voucher number of SK622/07.

Freshly purchased rhizomes were washed, sliced into small pieces, and allowed to air-dry overnight. The rhizome pieces were then ground in a commercial food processor (Cgoldenwall, Hangzou, China) into powder. Then, the powder was dissolved with hexane and water before the solution was repetitively subjected to hydrodistillation. Soluble oil was collected and water was removed. Crude essential oil was collected after the solvent had evaporated by using a rotary evaporator (Heidolph, Schwabach, Germany).

The crude essential oil was refrigerated at 4 °C for 48 h. Pure crystals that were formed were subjected to column chromatography (LiChro CART, Darmstadt, Germany). The purity of the eluate was determined by thin-layer chromatography (Merck, New York, NY, USA). Following repetitive recrystallization, zerumbone was stored at −80 °C until further use. High-performance liquid chromatography (HPLC) (Waters 2695, Pliening, Germany) analysis carried out on a sample of this batch of zerumbone showed 96.2% purity. Dimethyl sulfoxide (DMSO), Tween 20, and 0.9% NaCl at a ratio of 5:5:90 were used to dissolve zerumbone prior to treatment administration.

### 4.2. Drugs and Chemicals

Tetraethylammonium, glibenclamide, apamin, and charybdotoxin were purchased from Tocris (Bristol, UK). DMSO and Tween 20 were purchased from Sigma-Aldrich (St. Louis, MO, USA). Naloxone hydrochloride, β-funaltrexamine, naltrindole, nor-binaltorphimine, DMSO, and Tween 20 were bought from Sigma-Aldrich (St. Louis, MO, USA). Morphine sulphate was purchased from Lipomed (Cambridge, MA, USA). All drugs were dissolved in 0.9% NaCl. The vehicle consisted of DMSO, Tween 20, and 0.9% NaCl at a ratio of 5:5:90. All treatments were administered either intraperitoneally or subcutaneously at a volume of 10 mL/kg of body weight. Intraperitoneal injection was administered in the intraperitoneal cavity and subcutaneous administrations were made into the loose skin over the interscapular area.

### 4.3. Animals

Male ICR mice aged seven to eight weeks old (>25 g) were used in this study. Animals were randomly housed eight mice (n = 8) per cage for each treatment group at room temperature (24 ± 2 °C) and under standard environmental conditions of 12 h light and 12 h dark cycles. An acclimatization period of one week was allowed before the animals were subjected to tests. Standard laboratory feed and tap water was available ad libitum. All experimental procedures were evaluated and approved by the Institutional Animal Care and Use Committee (IACUC) of Universiti Putra Malaysia (reference number UPM/IACUC/AUP-R060/2013). All efforts to minimize the use of animals and pain caused to the animals were taken.

### 4.4. Induction of Neuropathic Pain

Neuropathic pain was induced by making constrictions to the sciatic nerve as previously described by Bennett and Xie [108], with slight modifications [109]. The entire surgical procedure was carried out under sterile conditions. First, the mice were anaesthetized with an intraperitoneal (i.p.) injection of tribromoethanol (250 mg/kg). The mid-thigh region of the hind limb was shaved before a small incision of approximately 5 mm was made to the skin. The biceps femoris muscle was separated by blunt dissection to expose the sciatic nerve. Three loose ligatures spaced 1 mm apart were made around the nerve proximal to the trifurcation using 4/0 silk sutures. Then, the incision to the skin was closed using a non-absorbable suture. Animals allocated in the sham group underwent the entire surgical procedure, however the sciatic nerves were not ligated. Povidone iodine was applied to the wound and animals were allowed to recover on flat paper bedding before they were returned to their home cages.

### 4.5. Experimental Design

On day 14 post-surgery, CCI-induced mice were pre-administered with either tetraethylammonium (a voltage-dependent K^+^ channel blocker, 4 mg/kg; i.p.), glibenclamide (an ATP-sensitive K^+^ channel blocker, 10 mg/kg; i.p.), apamin (a small-conductance Ca^2+^-activated K^+^ channel inhibitor, 0.04 mg/kg; i.p.), or charybdotoxin (a large-conductance Ca^2+^-activated K^+^ channel inhibitor 0.02 mg/kg; i.p.) 15 min prior to zerumbone (10 mg/kg; i.p.) treatment to determine the involvement of potassium channels in zerumbone-induced analgesia.

To determine the involvement of opioid receptors in zerumbone’s action, CCI-induced mice were pre-treated with NAL (a non-specific opioid receptor antagonist 10 mg/kg; i.p.) or NTI (a selective δ-opioid receptor antagonist, 20 mg/kg; s.c.), then 15 min before zerumbone (10 mg/kg; i.p.) treatment β-FN (a selective µ-opioid receptor antagonist; 40 mg/kg; s.c.) or nor-BNI (a selective κ-opioid receptor antagonist; 10 mg/kg; s.c.), was administered 24 h prior to zerumbone (10 mg/kg; i.p.) treatment. Morphine (10 mg/kg; i.p.) was used as the positive control in the NAL and β-FN groups [92].

Sham, vehicle, and zerumbone-only groups were administered treatments accordingly. The mice were subjected to nociceptive assays 30 min later.

### 4.6. Nociceptive Assays

Nociceptive assays were carried out via von Frey’s filament test followed by the Hargreaves test to evaluate the responses towards mechanical allodynia and thermal hyperalgesia. An interval of 5 min was allowed between assays

#### 4.6.1. VON Frey’s Filament Test

The treatment effects on the response towards mechanical allodynia were evaluated by von Frey’s filament test as described by Martinov et al. [110]. Briefly, the mice were allowed to acclimatize after they were placed in a Plexiglass chamber on an elevated wire mesh grid. Electronic von Frey’s anesthesiometer filaments (IITC Life Science Inc., Los Angeles, CA, USA) were applied to the middle dorsum of the ipsilateral paw of the mice when the animals were on all four limbs. The pressure at which the animals withdrew their paws from the filament was read from the automated reader. The mean of three readings was recorded as the withdrawal threshold. The cut-off point was set at 5 g within 20 s.

#### 4.6.2. Hargreaves Test

The treatment effects on the response towards thermal hyperalgesia were evaluated by the Hargreaves test as described by Hargreaves et al. [111]. Mice were allowed to acclimatize in a Plexiglass chamber placed on top of an elevated clear platform. Then, a radiant heat source from a Hargreaves apparatus (37370, UgoBasile, CA, USA) was directed to the mid-plantar surface of the ipsilateral paw when the animals were on all four limbs. The time taken for the mice to remove its paw from the heat source was recorded as the withdrawal latency. A cut off latency was set at 20 s, after which the heat was removed to prevent injury to the paws.

### 4.7. Rota Rod Test

In order to ensure that the behavioral responses exhibited by the animals were not due to the possible sedative effects of the treatments, the rota rod test was carried out. On day 14 post-CCI, the rota rod test was performed after 30 min in the zerumbone group and at the respective time points where nociceptive assays were carried out in the other groups. Each mouse was placed on a rota rod bar (UgoBasile, Gemonio, Italy) rotating at 20 rpm. The time each mouse spent on the rotating bar throughout a period of 3 min was recorded [26,28].

### 4.8. Statistical Analysis

Data are expressed as mean ± SEM. Statistical analysis was carried out using Statistical Analysis for Social Science (SPSS) version 16.0. Comparisons between groups were made with one-way ANOVA followed by Tukey’s post hoc test, where *p*-values of less than 0.05 were considered as significant.

## 5. Conclusions

We conclude that zerumbone’s antiallodynic and antihyperalgesic effects exhibited in the CCI-induced mice model of neuropathic pain involve K^+^ channels, specifically the K_V_, K_ATP_, BK_Ca_, and SK_Ca_ channels. Furthermore, zerumbone also involves the µ-, δ-, and κ-opioid receptor subtypes in its neuropathic pain modulation. Future research studies aiming to further investigate the other possible mechanisms of action are warranted in order to fully characterize the antineuropathic properties of zerumbone.
